# Association between multiple sclerosis and dietary patterns based on the traditional concept of food nature: a case-control study in Iran

**DOI:** 10.1186/s12883-021-02483-3

**Published:** 2021-11-18

**Authors:** Mohammad Hossein Sharifi, Parisa Keshani, Alireza Salehi, Amir Mohammad Jaladat, Zahra Mirzaei, Alireza Nikseresht

**Affiliations:** 1 Research Center for Traditional Medicine and History of Medicine, Shiraz University of Medical Sciences, Shiraz, Iran; 2 Health Policy Research Center, Institute of Health, Shiraz University of Medical Sciences, Shiraz, Iran; 3 Department of Persian Medicine, Shiraz University of Medical Sciences, Shiraz, Iran; 4 Department of Nutrition, School of Health, Qazvin University of Medical Sciences, Qazvin, Iran; 5 Department of Neurology, Shiraz University of Medical Sciences, Shiraz, Iran

**Keywords:** Multiple sclerosis, Dietary pattern, Food nature concept

## Abstract

**Introduction:**

It remains a matter of debate whether traditional concepts regarding the nature of food affect the development and progression of multiple sclerosis (MS).To date, there are limited studies that have investigated the association between MS and dietary patterns based on the categories of food nature (hot, cold, or balanced) defined in traditional medicine.

**Method:**

This case-control study was conducted from October 2019 to February 2020. In total, 60 patients diagnosed with MS within the preceding 6 months and referred to our neurology outpatient clinic were included in our case group. The control group included 180 patients who were referred to the same center for general or orthopedic surgery. Dietary intake was assessed in both groups through a reliable and valid semi-quantitative food frequency questionnaire. Data were assessed using principal component analysis.

**Results:**

The mean age of the participants was 44.9 ± 17.33 years. The analysis showed that four food patterns were distinguished (eigenvalue > 1), namely “additives and cold-natured foods”, “hot and balanced foods and nuts”, “dairy and legumes”, and “hot and balanced starches”. These food patterns explained 57.8% of the total variance.

After adjusting all confounding factors, individuals in the highest quartile and medium quartile of “additives and cold-natured foods” had an elevated MS risk compared with the lowest quartile (OR = 7.21, 95%CI = 2.01–12.38 and OR = 3.37, 95%CI = 1.02–11.35, respectively). Furthermore, individuals in the highest quartile of the “hot and balanced foods and nuts” group were protected against MS compared with its lowest quartile (OR = 0.28, 95%CI = 0.08–0.90). Moreover, a protective effect against MS was seen in the highest quartile of the “hot and balanced starches” group relative to its lowest quartile (OR = 0.34, 95%CI = 0.12–0.98). No significant association was found between “dairy and legumes” and the risk of MS.

**Conclusion:**

This study revealed that dietary patterns based on the traditional concept of food nature might be associated with the risk of developing MS. This represents the first work in this area, so further research is recommended.

## Introduction

It remains a matter of debate whether food patterns affect the development and progression of multiple sclerosis (MS) [1–3]. Several treatments for MS are now available, and addressing the diet is an important issue for both patients and healthcare practitioners [1]. Medications are prescribed to improve functioning during an attack and prevent new MS attacks or improve signs and symptoms [4, 5]. These medications are modestly effective, have side effects, and are sometimes poorly tolerated [4, 6]. In addition, evidence-based dietary guidelines for MS patients do not exist [3, 7]. Scientific evidence indicates that dietary factors can exacerbate or improve symptoms of MS through different mechanisms (metabolic, inflammatory, etc.) in both primary-progressive MS (PPMS) and relapsing-remitting MS (RRMS) [8]. Clear and effective dietary strategies might help modify or slow the disease course, treat relapses, manage symptoms, improve function and safety, and enhance emotional health.

MS is one of the leading causes of neurological disability in young adults, and its onset is usually seen in the third or fourth decade of life [9, 10]. MS has a substantial influence on individual quality of life and places a large economic burden on society [11]. While its cause is unclear, the underlying mechanism is thought to be either the malfunction of the immune system or the failure of myelin-producing cells [4]. Proposed causes for this include genetic and environmental factors. Evidence shows that dietary factors and nutrients such as vitamins D and B12 can influence the immune system through various mechanisms [12–14]. Dietary modifications are also considered complementary treatment for controlling MS [15]. Recent studies have revealed that dietary interventions such as following an anti-inflammatory diet and consuming appropriate dietary supplements minimize the pro-inflammatory factors and can improve the efficacy of immunomodulatory drugs, enhancing the well-being of MS patients [12, 16].

Mizaj, or temperament, is a basic concept in Persian medicine which classifies persons and their surrounding affecting factors based on the main four qualities of hot, cold, dry, and wet. In this concept, each person has a dominant temperament that is present in his physical and mental characteristics and can justify his reactions to different environmental factors with different qualities, including climate change and nutrition [17, 18]. Dietary pattern analysis is a scientific method for understanding the basis of eating behaviors and nutrition-related diseases [19]. Based on traditional Persian medicine (TPM), foods can be categorized as having a hot, cold, or balanced nature [20]. The concept of food nature is known not only in TPM, but also in the traditional medicine of the Indian, European, Arabic, Roman, Greek, and Chinese cultures [21].The concept of natural food, however, is not entirely intuitive for word scientists, but research in this field is growing. Recently, some studies have analyzed foods based on this concept and have revealed that the nutrients of foods may be one of the distinguishing factors for categorizing their cold-hot properties [22, 23]. A previous study showed that hot-natured foods have beneficial effects on improving the clinical score of MS through an immunomodulatory mechanism [24]. However, another study showed that cold and hot-natured foods do not impact brain health or behavior. There is a societal belief that consuming foods of a certain nature can increase the risk of the development and progression of MS. Some people claim that cold-natured foods, such as milk or beef, can increase the risk of developing diseases. Nonetheless, little scientific evidence is available in this regard, and more accurate estimates of valuable predictors in MS are needed, including qualitative markers or specific dietary patterns [25].

Food pattern analysis utilizes a more scientific approach than food or nutrient intake evaluation for understanding the association between diet and disease. To date, a limited number of studies have investigated the association between MS and dietary patterns based on the traditional concept of food nature (hot, cold, or balanced). This study, therefore, was conducted to assess the relationship between MS and the nature of various foods through factor analysis.

## Method

### Study participants

**Fig. 1:**
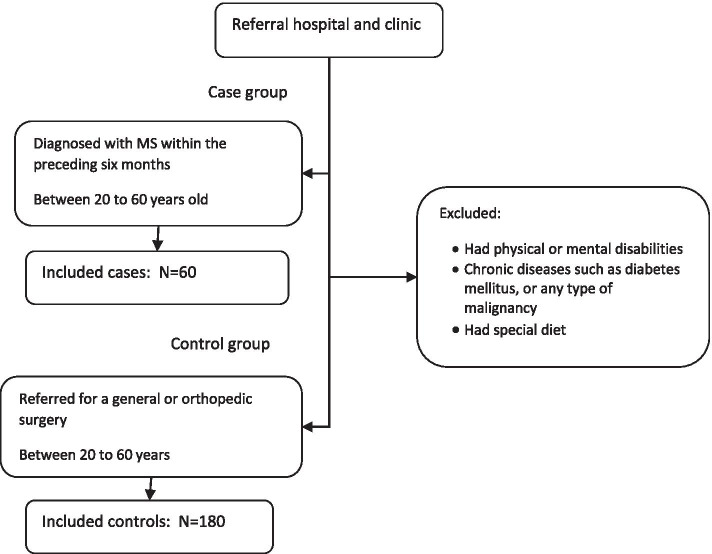
Diagram to show case selection and inclusion/exclusion criteria

This case-control study was conducted from October 2019 to February 2020. The case group comprised 60 patients aged between 20 to 60 years who were diagnosed with MS within the preceding 6 months (without an identified pattern of MS) and referred to our neurology outpatient clinic or ward for a check-up or to receive beta interferon. The control group was composed of 180 patients with a similar age range who referred to the same center for general or orthopedic surgery. These study subjects were not under a special diet and had no non-communicable diseases. Individuals with physical or mental disabilities, chronic diseases such as diabetes mellitus, or any type of malignancy were excluded. Figure 1 shows the details.

A socio-demographic questionnaire was used to gather the participants’ data, including marital status, occupational status, education level, gender, age, and smoking habit. All anthropometric measurements were obtained by the same interviewer. A tape measure was used to determine the participants’ height to the nearest 0.1 cm while they were barefooted and maintained a standing position with their backs against the wall. Weight was measured and documented to the nearest 0.5 kg using a digital scale while the participants were wearing light clothes and no shoes; the BMI was hence calculated.

### Dietary assessment

The typical dietary intake of each subject was assessed through a reliable and valid semi-quantitative food frequency questionnaire (FFQ) consisting of 168 items [26]. The standard serving size that is normally consumed by Iranians was considered. Participants were asked to record their intake level on a regular, weekly, monthly, or annual basis of an expected serving of each food item. The chosen frequency classification for each food item was then converted into a daily intake. Each food item in the validated FFQ was evaluated based on its traditional concept of food nature, such as their cold, hot, or balanced nature, by three TPM experts. Inter-rater reliability was measured by Cohen’s kappa coefficient with a substantial degree of agreement (0.68) being observed between the experts.

**Table 1:** Food items based on the validated Iranian food frequency questionnaire, categorized into food groups according to their nature

	Food groups	Foods items
1	**Cold-natured meats**	Cow and veal meat, hen and chicken, fish and seafood, canned tuna, hamburgers, brain-sausages, bologna, and pizza.
2	**Additives**	Chips, cheese balls, sugar cubes, sugar, honey, jam, cola, traditional sweet snacks, chocolate, sugarplum, candy, halva, pepper, chili sauce, salted vegetables, pickles, salt, condiments, cake, confectionery, biscuits, and crackers.
3	**Cold-natured fruits**	Watermelon, apricot, cherries, peaches, nectarine, greengages, grapefruit, oranges, tangerines, pomegranates, plums, and strawberries.
4	**Fats**	Butter, olive oil, animal fat, margarine, and hydrogenated vegetable oil.
5	**Acidic seasonings**	Lime, lemon juice, and vinegar
	**Nuts and dried fruits**	Dried fig, date, resin, dried berries, dried apricot, dried plum; all nuts (pistachio, almond, walnuts, hazelnuts, peanuts, etc.)
6	**Hot-natured meats**	Sheep meat, eggs, and organ meats (tongue, rumen, face meat, foot, heart, and liver)
7	**Hot and balanced fruits**	Pear, apple, fresh berries, cantaloupe, melon, fig, grapes, kiwifruit, persimmon, banana, fruit juices, and compote.
8	**Legumes**	Lentil, soya bean, peas, split peas, and all beans.
9	**Cold-natured starches**	Baguette bread, toast, spaghetti, vermicelli, barley, potato, and broad beans
10	**Dairy**	Milk (low fat and high fat), yogurt (low fat and high fat), condensed yogurt, yogurt drink, icecream, cheese curds, creamy cheese, and cream.
11	**Hot and balanced starches**	Traditional bread (*Lavash, Barbari, Sangak, Taftoon*), noodles, wheat, rice, flour, bulgur, pumpkin, green peas, and corn.

**Fig. 2:**
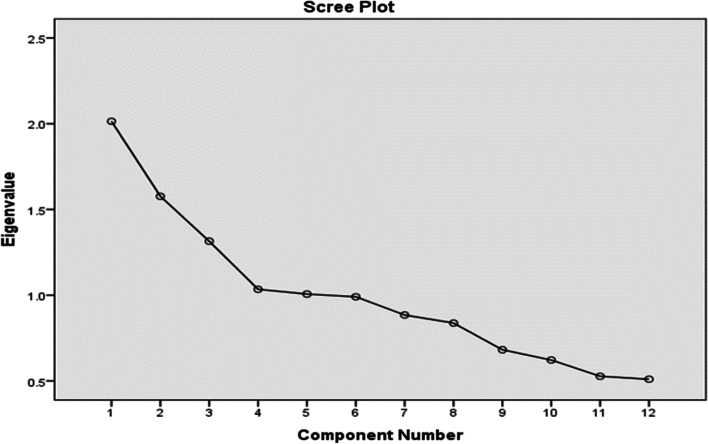
Screen plot for food patterns

### Personal *Mizaj* questionnaire

In this study, the Mojahedi Mizaj Questionnaire (MMQ), a self-reported questionnaire for indicating personal *Mizaj*, was used; its validity and reliability have been shown in previous studies [27]. This short questionnaire contains 10 questions that are scored from 1 to 3. The total score of the first eight questions can represent a hot (≥19) or cold (≤14) *Mizaj*, while the total score of the two remaining questions is indicative of wet (≥5) or dry (≤3) *Mizaj*.

### Statistical analysis

Each food item in the validated FFQ was evaluated based on its traditional concept of food nature, such as their cold, hot, or balanced nature, by three TPM experts. Inter-rater reliability was measured by Cohen’s kappa coefficient with a substantial degree of agreement (0.68) being observed between the experts. Based on food nature, the food items were categorized into 12 food groups (Table 1) and principal component analysis was used to determine the leading dietary trends. Four food patterns were distinguished based on their eigenvalues (> 1) and scree plot analysis (Fig. 2).

Principle component analysis was used to identify the dietary trends. For further analysis, only variables with an eigenvalue of 1.0 or more were kept. Orthogonal rotation (varimax) was introduced to simplify the analysis of the results. The analysis involved food groups with a factor loading of ≥ ± 0.4. The trends were named according to the highest factor loadings on each pattern. The dietary habits were eventually categorized into quartiles with the first quartile displaying low intake and the third showing high adherence to the dietary pattern.

Using multivariable logistic regression, the association between the risk of MS (dichotomous: Yes/No) as a dependent variable and the quartiles of four dietary trends was assessed and reported by odds ratios (ORs) and 95% confidence intervals (CIs). Variables with *p*-values ≤0.2 in univariable analysis were entered into the models. In this respect, three logistic regression models were tested: Model 1 was unadjusted; Model 2 was adjusted for BMI, energy intake, gender (male, female), and age; Model 3 was optimized for the patients’ *Mizaj* (hot, balanced, cold) in addition to variables entered in previous models.

SPSS version 24 was used to analyze the collected data. The t-test was utilized for comparing normally distributed quantitative variables, while qualitative variables were compared using the chi-square test.

## Results

Table 2 shows the participants’ demographic characteristics, anthropometrics, *Mizaj* status, and dietary intake. Dietary information on three of the control group members was excluded, because their energy intake was over-reported. Case and control groups were not significantly different based on gender, marital status, *Mizaj* status, BMI, or energy, carbohydrate, fat, and protein intake. However, smoking was more common (*p* = 0.02) and the mean age was higher (*p* < 0.001) in the control group.

**Table 2:** Demographic and clinical characteristics of the participants

	Control ***n*** = 177	Case ***n*** = 60	***P***-value
	**(n %)**	**(n %)**	
**Gender**			0.21
Female	134 (75.7)	50 (82.0)	
Male	43 (24.3)	11 (18.0)	
**Marital status**			0.27
Single	39 (22)	19 (31.1)	
Married	136 (76.8)	42 (68.9)	
Divorced	2 (1.1)	–	
**Smoking**			0.02
Yes	34 (19.2)	7 (13.2)	
No	143 (80.8)	53 (86.8)	
**Personal*Mizaj*(Hot or Cold)**			0.19
Hot	44 (24.9)	15 (24.6)	
Balanced	90 (50.8)	26 (42.6)	
Cold	43 (24.3)	20 (32.8)	
**Personal*Mizaj*(Wet or Dry)**			0.92
Dry	69 (39.0)	23 (37.7)	
Balanced	52 (29.4)	17 (27.9)	
Wet	56 (31.6)	21 (34.4)	
	**Mean ± SD**	**Mean ± SD**	
**Age (years)**	44.9 ± 17.3	33.6 ± 10.8	< 0.001
**BMI (kg/m**^**2**^**)**	26.2 ± 4.3	25.1 ± 4.2	0.15
**Energy intake (kcal)**	2569.4 ± 744.5	2438.3 ± 640.2	0.22
**Carbohydrate intake (gr)**	417.1 ± 130.9	405.3 ± 110.0	0.52
**Fat intake (gr)**	67.6 ± 31.0	63.5 ± 21.7	0.34
**Protein intake (gr)**	88.3 ± 31.6	81.6 ± 25.5	0.13

Four dietary patterns were extracted based on their traditional concept of food nature. The first dietary pattern was named “additives and cold-natured foods” and identified by high intakes of cold-natured meats, cold-natured fruits, fats, additives, and acidic seasonings. The second pattern was named “hot and balanced foods and nuts” and distinguished by a high intake of nuts and dried fruits, hot-natured meats, and hot-natured fruits. The third food pattern was labeled as “dairy and legumes” and characterized by high amounts of legumes, cold-natured starches, and dairy products. The fourth pattern was named “hot and balanced starches” and characterized by high amounts of balanced starchy products. The list of food groups and their factor loadings are presented in Table 3. These dietary patterns explained 57.81% of the total variance. Table 4 indicates the association between the quartiles of dietary patterns and the risk of MS. After adjusting for all confounding factors (Model 3), individuals in the highest and middle quartiles of “additives and cold-natured foods” were found to have an elevated MS risk compared to those in the lowest quartile (OR = 7.21, 95% CI = 2.01–12.38 and OR = 3.37, 95% CI = 1.02–11.35, respectively), with this association being significant (*p* = 0.002). Conversely, individuals in the highest quartile of the “hot and balanced foods and nuts” group were protected against MS compared with its lowest quartile (OR = 0.28, 95% CI = 0.08–0.90). This association was also significant (*p* = 0.03). Furthermore, a protective effect against MS was seen in the highest quartile of the “hot and balanced starches” group relative to its lowest quartile (OR = 0.34, 95% CI = 0.12–0.98); again, this association was significant (*p* = 0.04). Finally, no significant association was found between the “dairy and legumes” dietary pattern and the risk of MS. Based on Akaike criterion (AIC), the third model was considered as the best of all models in this study.

**Table 3:** Rotated factor loading matrix for the four food patterns

	Additives and cold-natured foods	Hot and balanced foods and nuts	Dairy and legumes	Hot and balanced starches
**Cold-natured meats**	0.43			
**Cold-natured fruits**	0.52			
**Fats**	0.50			
**Additives**	0.63			
**Acidic seasonings**	0.54			
**Hot and balanced meats**		0.47		
**Hot and balanced fruits**		0.47		
**Nuts and dried fruits**		0.51		
**Dairy**			−0.62	
**Legumes**			0.58	
**Cold-natured starches**			0.43	
**Hot and balanced starches**				0.65

**Table 4:** Odds ratios and 95% confidence intervals for the association between dietarypatterns and MS

Dietary pattern	Q1	Q2	Q3	Q4	***P***-value
**Additives and cold-natured foods**					
Model1	1	2.34 (0.88–6.20)	2.15 (0.78–5.89)	3.02 (1.14–8.02)	0.02
Model2	1	2.37 (0.77–7.26)	3.07 (0.93–10.08)	6.87 (2.00–10.53)	0.002
Model3	1	2.42 (0.77–7.57)	3.37 (1.02–11-35)	7.21 (2.01–12.38)	0.002
**Hot and balanced foods and nuts**					
Model1	1	0.78 (0.33–1.84)	0.99 (0.42–2.30)	0.36 (0.14–0.94)	0.03
Model2	1	0.73 (0.27–1.93)	0.84 (0.32–2.19)	0.28 (0.09–0.88)	0.03
Model3	1	0.70 (0.26–1.90)	0.76 (0.28–2.05)	0.28 (0.08–0.90)	0.03
**Dairy and Legumes**					
Model1	1	1.18 (0.48–2.92)	1.59 (0.66–3.82)	0.59 (0.22–1.54)	0.20
Model2	1	0.89 (0.31–2.53)	1.31 (0.46–3.74)	0.32 (0.09–1.16)	0.07
Model3	1	0.87 (0.30–2.54)	1.26 (0.43–3.70)	0.30 (0.08–1.13)	0.07
**Hot and balanced starches**					
Model1	1	0.54 (0.23–1.28)	0.92 (0.40–2.10)	0.33 (0.13–0.84)	0.02
Model2	1	0.58 (0.22–1.50)	1.01 (0.40–2.55)	0.38 (0.13–1.03)	0.06
Model3	1	0.56 (0.21–1.52)	0.97 (0.37–2.50)	0.34 (0.12–0.98)	0.04

Three logistic regression models were tested: Model 1 was unadjusted; Model 2 was adjusted for BMI, energy intake, gender, and age; Model 3 was further optimized for the patients’ Mizaj (Hot, Balanced, Cold)

## Discussion

The present study is the first to analyze dietary patterns among MS patients and controls based on the traditional concept of food nature. As reported above, four major dietary patterns were recognized, explaining 57.8% of the total variance. This study showed that cold-natured foods, high-fat diets, additives, and acidic seasonings might elevate the risk of MS, whereas hot and balanced foods, fruits, starches, and nuts might offer protection against the disease. The dairy and legumes food pattern showed no significant association with MS.

Recently, contradictory findings have emerged about the impact of food patterns and diet quality on MS. A systematic review on the influence of diet on MS indicated that deficiencies in micronutrients (vitamin D and vitamin B12) can influence the progression of MS [15]. Mitochondrial dysfunction, epigenetic modification, gut microbiota, and neuroinflammation are major pathways that are accountable for food action on brain health [28, 29]. A previous study indicated that dietary antioxidants and abnormalities in lipid and glucose metabolism may influence the progression of neurodegenerative diseases [30, 31]. Healthy eating patterns, such as a low-fat or Mediterranean diet, may reduce systemic inflammation and MS attacks, thereby improving the quality of life [16]. However, another previous study showed that there is no evidence of a correlation between diet quality and the risk of developing MS among women [32]. Nonetheless, maintaining a healthy diet (high in poultry, fish, eggs, vegetables, and legumes) instead of a Western diet (high in meat, full-fat dairy; low in whole grains, nuts, fresh fruit, and low-fat dairy) may be beneficial for those at high risk of MS [33]. Hence, further research into the relationships between dietary patterns and MS seems warranted.

The theory of “hot and cold natures” is based on the beliefs of Hippocrates (Greek physician, 460—375 BC) and Galen (199—129 BC) [24, 34]. A recent study found that cold- or hot-natured foods did not affect the brain health or behavior of students [35]. A study by Chunhong Liu et al. evaluated 284 foods according to their cold or hot nature, suggesting that the nutrients of foods could be one of the distinguishing factors for categorizing their cold or hot essence [22]. Another multivariate analysis found that 18 food components had major effects on the cold or hot properties of foods [23].

In this study, cold-natured foods included cold-natured meats (cow and veal meat, hen and chicken, fish/seafood, canned tuna, hamburgers, sausages, bologna, and pizza), cold-natured fruits (watermelon, apricot, cherries, peach, nectarine, greengage, grapefruit, orange, tangerine, pomegranate, plum, and strawberry), and additives (chips, cheese balls, sugar, salt, etc.). The analyses in the current study showed that food patterns that include foods with a cold-nature, a high-fat diet, additives, and acidic seasoning may increase the risk of MS. The results of this study indicated that a high-fat diet (butter, olive oil, animal fat, margarine, hydrogenated vegetable oil) could increase the risk of MS. In line with this finding, a previous study showed that animal fat can affect the progression of MS through the mechanism of low-grade systemic inflammation [36]. The current results are also in accord with the authors’ earlier observations, which showed that animal fat may increase the risk of MS [20]. However, this data must be interpreted with caution, because saturated fatty acids, monounsaturated fatty acids, and multi-unsaturated fatty acids were not distinguished in our analysis. The current study showed that some cold-natured foods can increase the risk of MS. The exploration of potential mechanisms is difficult, but recent Chinese studies have shown that low levels of antioxidants or vitamins (B6; folate) may contribute to the cold essence of certain foods [23, 37]. This finding is in agreement with recent studies indicating that deficiencies in folate, vitamin B12, and other vitamins might contribute to the progression of MS.

The current study showed that food patterns that include hot-natured meats or fruits, starches, and nuts with a hot and balanced nature might be protective against the development of MS. Here, hot-natured foods included sheep meat, eggs, and organ meats (tongue, rumen, face meat, foot, heart, and liver), while hot and balanced fruits consisted of pears, apples, fresh berries, cantaloupe, melon, fig, grapes, kiwifruit, persimmon, banana, fruit juices, and compote. Consistent with this study, in a double-blind randomized trial, a hot-natured diet had beneficial effects on improvement in the clinical scores and immunological indicators (IL-4, IFN- and IL-17) of 100 MS patients [24]. Another possible explanation for this is that the total antioxidant capacity of the hot-natured diet is higher relative to the cold-natured diet [38]. This finding is in part contradictory to previous studies that suggested a high intake of unprocessed red meat may decrease the risk of MS, though no distinction was made between sheep meat and cow meat in these studies [20, 39]. Additionally, research has shown that the restriction of dietary red meat has no major effect on the severity of the disease [40, 41].

In the current study, based on theories of traditional medicine, fruits were categorized according to their cold or hot nature, and the results showed that hot-natured fruits had a protective role, whereas cold-natured fruits increased the risk of MS. However, the findings of the current study do not completely support the previously established literature. Abdollahpour et al. indicated that fruit and vegetable intake might serve as a protective factor against MS [41]. Furthermore, in other studies, high fruit and vegetable intake was linked with a reduced risk of MS [8, 42]; a possible explanation for this may be that fruits and vegetables are rich in vitamins B and C [43, 44]. As mentioned, cold-natured fruits increase the risk of MS. This partial inconsistency may be due to the fact that hot- and cold-natured fruits were not separated in the previous studies. Generally, nuts have a hot nature according to TPM and fulfill a protective role against MS. Based on previous studies, nuts are rich in minerals and omega-3; therefore, they could have an anti-inflammatory effect on pathways of inflammation and MS [8, 45].

The strong point of the analysis in the current study is the use of the FFQ. Due to the limitation in the number of cases, however, a case-control design with three-times more controls was utilized to improve the validation of analyses. In addition, based on the design of study, these studies cannot prove causality, but they can provide strong evidence and strength association. To develop a full picture of dietary patterns based on the traditional concept of food nature, powerful research methods, such as clinical trial studies, will be needed.

## Conclusion

The effect of food and diet on the development and progression of MS remains a subject of debate. The evaluation of dietary patterns in patients with MS could provide scientific evidence in response to this ongoing debate. The current study showed that cold-natured foods, high-fat diets, and a high intake of additives and acidic seasonings might be associated with an elevated risk of MS and lower quality of life. Conversely, hot-natured foods and hot and balanced fruits, starches, and nuts might be protective against MS. The association of healthy dietary habits with quality of life has been recognized in several studies. Based on the current results, it is possible that a protective dietary pattern could affect MS progression and improve physical and mental well-being and quality of life. This research is the first in its area, so the findings need to be interpreted with caution. Further research is required to achieve a detailed understanding of the relationship between consumption of hot- or cold-natured foods and MS development and progression.

## Data Availability

The datasets used and/or analyzed during the current investigation are available upon reasonable request from the corresponding author.
